# Loperamide, pimozide, and STF-62247 trigger autophagy-dependent cell death in glioblastoma cells

**DOI:** 10.1038/s41419-018-1003-1

**Published:** 2018-09-24

**Authors:** Svenja Zielke, Nina Meyer, Muriel Mari, Khalil Abou-El-Ardat, Fulvio Reggiori, Sjoerd J. L. van Wijk, Donat Kögel, Simone Fulda

**Affiliations:** 1 Institute for Experimental Cancer Research in Pediatrics, Goethe-University Frankfurt, Komturstr. 3a, 60528 Frankfurt, Germany; 2 Experimental Neurosurgery, Goethe-University Hospital, Theodor-Stern-Kai 7, 60590 Frankfurt, Germany; 3 Department of Cell Biology, University of Groningen, University Medical Center Groningen, A. Deusinglaan 1, 9713 AV Groningen, Netherlands; 4 Department of Medicine II, Hematology/Oncology, Goethe University, Theodor-Stern-Kai 7, 60590 Frankfurt, Germany; 5 German Cancer Consortium (DKTK), Partner Site Frankfurt, Frankfurt, Germany; 6 German Cancer Research Center (DKFZ), Heidelberg, Germany

## Abstract

Autophagy is a well-described degradation mechanism that promotes cell survival upon nutrient starvation and other forms of cellular stresses. In addition, there is growing evidence showing that autophagy can exert a lethal function via autophagic cell death (ACD). As ACD has been implicated in apoptosis-resistant glioblastoma (GBM), there is a high medical need for identifying novel ACD-inducing drugs. Therefore, we screened a library containing 70 autophagy-inducing compounds to induce ATG5-dependent cell death in human MZ-54 GBM cells. Here, we identified three compounds, i.e. loperamide, pimozide, and STF-62247 that significantly induce cell death in several GBM cell lines compared to CRISPR/Cas9-generated ATG5- or ATG7-deficient cells, pointing to a death-promoting role of autophagy. Further cell death analyses conducted using pharmacological inhibitors revealed that apoptosis, ferroptosis, and necroptosis only play minor roles in loperamide-, pimozide- or STF-62247-induced cell death. Intriguingly, these three compounds induce massive lipidation of the autophagy marker protein LC3B as well as the formation of LC3B puncta, which are characteristic of autophagy. Furthermore, loperamide, pimozide, and STF-62247 enhance the autophagic flux in parental MZ-54 cells, but not in *ATG5* or *ATG7* knockout (KO) MZ-54 cells. In addition, loperamide- and pimozide-treated cells display a massive formation of autophagosomes and autolysosomes at the ultrastructural level. Finally, stimulation of autophagy by all three compounds is accompanied by dephosphorylation of mammalian target of rapamycin complex 1 (mTORC1), a well-known negative regulator of autophagy. In summary, our results indicate that loperamide, pimozide, and STF-62247 induce ATG5- and ATG7*-*dependent cell death in GBM cells, which is preceded by a massive induction of autophagy. These findings emphasize the lethal function and potential clinical relevance of hyperactivated autophagy in GBM.

## Introduction

GBM represents the most aggressive malignant primary brain tumor with a median survival of 16 months after radio-chemotherapy^1,2^. Importantly, GBMs were shown to be highly resistant to caspase-dependent apoptosis^3,4^. As defects in apoptosis signaling contribute to tumorigenesis and chemoresistance, there is a high medical need for novel therapies^5^. Therefore, the induction of alternative forms of cell death, such as ACD has emerged as an attractive concept to trigger cell death in GBM^6^.

Macroautophagy (hereafter autophagy) is a catabolic process that involves the degradation of cytoplasmic components, including damaged organelles and protein aggregates in double-membraned autophagosomes that eventually fuse with lysosomes to allow cargo degradation^7^. To date, numerous autophagy-related (ATG) proteins have been characterized in yeast, many of which have known orthologs in mammals^8^. ATG proteins are required for virtually every step of autophagy and autophagosome biogenesis, starting with nucleation of the initial autophagosome precursor, the phagophore^9^. Subsequent membrane expansion, closure of the autophagosome as well as intralysosomal degradation depend on the concerted action of two ubiquitin-like conjugation systems, which include ATG5 and ATG7^10,11^. Well-described marker proteins for monitoring autophagy progression are members of the LC3/GABARAP protein family^12^. Soluble LC3/GABARAP is constitutively processed by ATG4 proteases and, upon onset of autophagy, becomes conjugated to the phosphatidylethanolamine (PE) present in autophagosomal membranes through the action of the two ubiquitin-like conjugation systems^8,13^.

The cellular outcome of autophagy induction is highly contextual. On the one hand, it is well-described that autophagy serves to adapt to stressful conditions, such as nutrient deprivation, oxidative damage or accumulation of misfolded proteins and, thus, promotes cellular survival^14–16^. On the other hand, there is growing evidence suggesting a cell death-promoting role of autophagy, referred to as type II cell death or ACD^17^. Enforced hyperactivation of autophagy has been described to trigger massive cellular self-digestion beyond the point of allowing cellular survival^18,19^. Moreover, autophagosomes can serve as signaling platforms that facilitate the activation and integration of different cell death pathways, such as necroptosis, through caspase-8 activation on autophagosomal membranes^20^. Furthermore, selective degradation of specific proteins, like the reactive oxygen species (ROS) scavenger catalase, can induce ACD as well^21^.

Several key criteria have been defined for bona fide ACD. First of all, the term ACD should be limited to cases of cell death that can be suppressed through either genetic or pharmacological inhibition of at least two members of the autophagic core machinery^22^. Second, the death process should be mediated via an enhanced autophagic flux instead of blocking autophagy at any of its stages^23^.

Intriguingly, in vitro and in vivo induction of ACD has been investigated as a potential therapeutic approach in apoptosis-resistant cancers^24–26^. Hence, the identification of novel ACD-inducing drugs in highly malignant GBM cells remains a promising strategy. In order to identify novel inducers of ACD, we screened a compound library containing 70 known autophagy-inducing drugs on parental as well as ATG5-deficient MZ-54 GBM cells.

## Results

### Loperamide, pimozide, and STF-62247 induce autophagy-dependent cell death in GBM cells

**Fig. 1:**
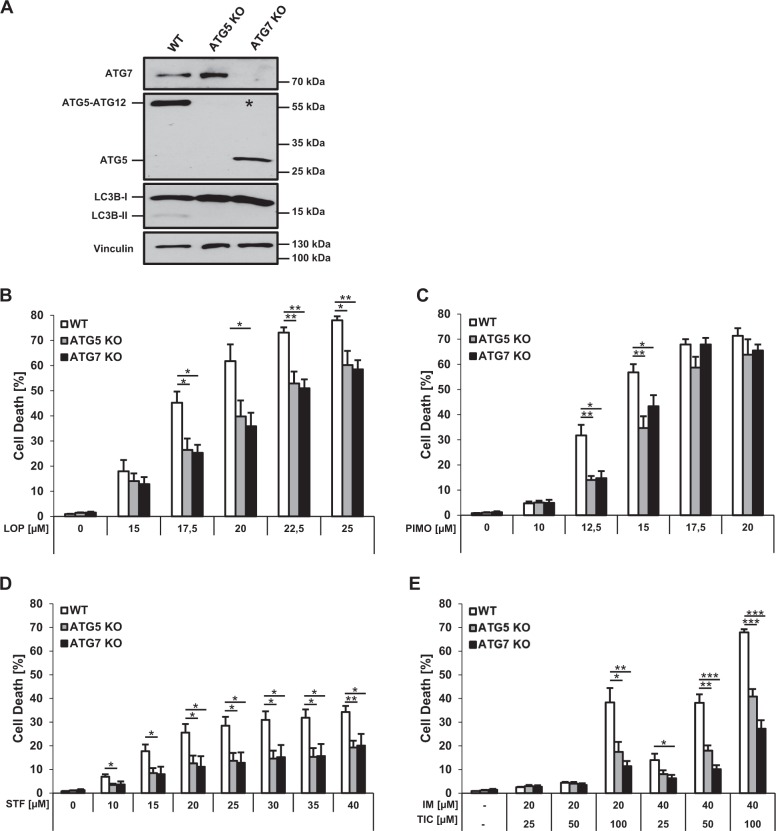
Loperamide, pimozide, and STF-62247 induce autophagy-dependent cell death in GBM cells. **a** Lysates from untreated MZ-54 WT, *ATG5*, and *ATG7* KO cells were subjected to Western blotting with the indicated antibodies and vinculin as loading control. The asterisk indicates the absence of the ATG5-ATG12 conjugate in *ATG7* KO cells. **b–d** MZ-54 WT, *ATG5* KO, and *ATG7* KO cells were treated with indicated concentrations of loperamide, pimozide, STF-62247, and IM/TIC for 48 h. Cell death was assessed by measuring the PI uptake as fraction of total nuclei determined by Hoechst counterstaining using high-content fluorescence microscopy. Data are presented as mean and SEM of 3−5 independent experiments performed in triplicate. Significances are calculated against WT cells treated with the same drug concentration. *p < 0.05, **p < 0.01, ***p < 0.001. UT untreated, LOP loperamide, PIMO pimozide, STF STF-62247, IM imipramine hydrochloride, TIC ticlopidine

To identify novel inducers of ACD we screened the Enzo Screen-Well™ library containing 70 known autophagy-inducing drugs for cell death induction in wild-type (WT) MZ-54 GBM cells and *ATG5* or *ATG7* KO MZ-54 cells. Using next-generation sequencing we identified the heterozygous gain-of-function mutation ENSP00000391127:p.Arg248Trp within the *TP53* gene of MZ-54 cells, which has been reported to render cells less sensitive towards apoptosis-inducing drugs^27,28^. We previously described the generation of CRISPR/Cas9 *ATG5* KO cells derived from the MZ-54 cell line^29^ (Fig. 1a). Of note, the ATG5-ATG12 conjugate was found to be absent not only in *ATG5* KO, but also in *ATG7* KO cells (depicted by asterisk), which is in line with the notion that ATG7 is required for the conjugation of ATG12 to ATG5 during autophagosome maturation^30^. Importantly, among the tested compounds we identified loperamide, pimozide, and STF-62247 to induce ATG5- and ATG7-dependent cell death in MZ-54 cells at various concentrations, as loperamide-, pimozide- or STF-62247-triggered cell death was significantly reduced in *ATG5* or *ATG7* KO compared to control cells (Fig. 1b–d). As a positive control, we used the antidepressant drug imipramine hydrochloride (IM) in combination with the anticoagulant drug ticlopidine (TIC), since this combination has previously been reported to induce ACD in GBM cells^24^. As expected, treatment with IM and TIC triggered cell death in a concentration-dependent manner in parental MZ-54 cells, which was significantly decreased in *ATG5* or *ATG7* KO MZ-54 cells (Fig. 1e). As a negative control, treating MZ-54 cells with the apoptosis-inducing compound ABT-737 and etoposide induced cell death in WT MZ-54 cells to a similar extent as in *ATG5* or *ATG7* KO cells (Suppl. Fig. S1)^31^.

**Fig. 2:**
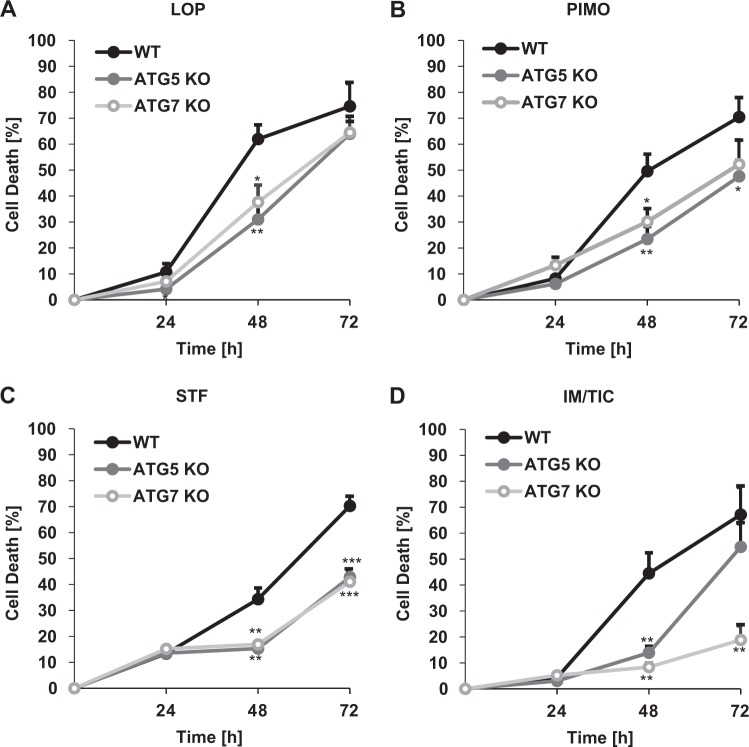
Loperamide, pimozide, and STF-62247 induce autophagy-dependent cell death of MZ-54 in a time-dependent manner. **a–d** MZ-54 cells were treated with 17.5 µM loperamide, 15 µM pimozide, 40 µM STF-62247, and 20 µM IM/100 µM TIC for 24, 48, and 72 h. Cell death was assessed by measuring the PI uptake as fraction of total nuclei determined by Hoechst counterstaining using high-content fluorescence microscopy. Mean and SEM of 3−5 independent experiments performed in triplicate are shown. Significances are calculated versus WT cells. *p < 0.05, **p < 0.01, ***p < 0.001. LOP loperamide, PIMO pimozide, STF STF-62247, IM imipramine hydrochloride, TIC ticlopidine

Kinetic analysis showed that all compounds induced cell death in a time-dependent manner (Fig. 2a–d, Suppl. Fig. S2). KO of *ATG5* or *ATG7* protected cells from loperamide-, pimozide- and IM/TIC-induced cell death after 48 h and from STF-62247-induced cell death after 48 h as well as 72 h (Fig. 2a–d).

Together, these findings indicate that autophagy contributes to cell death induced by loperamide, pimozide, and STF-62247, similarly to IM/TIC.

To investigate whether the induction of autophagy-dependent cell death by loperamide, pimozide, and STF-62247 also occurs in other GBM cell lines we extended our experiments to additional GBM cell lines. Importantly, loperamide-, pimozide- and STF-62247-induced cell death was significantly reduced in *ATG5* or *ATG7* KO LN-229 or U343 GOS-3 cells compared to the corresponding parental cell lines (Suppl. Fig. S3). This underscores that loperamide, pimozide, and STF-62247 can induce autophagy-dependent cell death in GBM cells.

### Loperamide-, pimozide- or STF-62247-induced cell death does not primarily involve apoptosis, ferroptosis or necroptosis

**Fig. 3:**
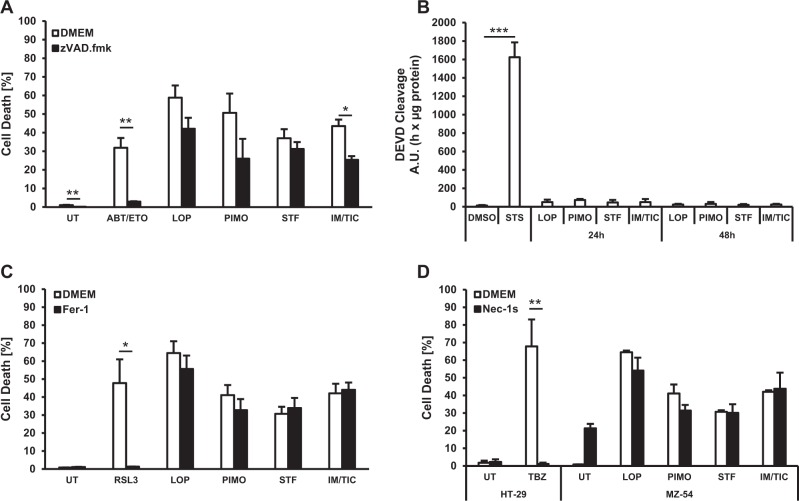
Loperamide, pimozide- or STF-62247-induced cell death does not primarily involve apoptosis, ferroptosis, or necroptosis. **a**, **c**, **d** MZ-54 cells were pretreated for 1 h with 20 µM zVAD.fmk (**a**), 5 µM Fer-1 (**c**) or 30 µM Nec-1s (**d**) followed by treatment with 17.5 µM loperamide, 15 µM pimozide, 40 µM STF-62247, 20 µM IM/100 µM TIC, 25 µM ABT-737/100 µM etoposide, 500 nM RSL3 or 1 ng/mL TNF${\alpha}$ + 0.5 µM BV6 for 48 h. Cell death was assessed by measuring the PI uptake as fraction of total nuclei determined by Hoechst counterstaining using high-content fluorescence microscopy. HT-29 cells served as positive control for induction of necroptotic cell death. **b** MZ-54 cells were treated with 3 µM STS, 15 µM loperamide, 15 µM pimozide, 40 µM STF-62247, or 20 µM IM/100 µM TIC for the indicated time points. Caspase-3 activity was determined by quantifying alterations in Ac-DEVD-AMC fluorescence. Mean and SEM of 3−4 independent experiments performed in triplicate are shown. *p < 0.05, **p < 0.01, ***p < 0.001. UT untreated, LOP loperamide, PIMO pimozide, STF STF-62247, IM imipramine hydrochloride, TIC ticlopidine, STS staurosporine

To further understand the type of cell death induced by exposing MZ-54 cells to loperamide, pimozide, STF-62247, or IM/TIC cell death was assessed in the absence or presence of pharmacological inhibitors of apoptosis, ferroptosis, and necroptosis. Addition of the broad-range caspase inhibitor zVAD.fmk failed to protect MZ-54 cells from loperamide-, pimozide- or STF-62247-induced cell death and only partially rescued cells from IM/TIC-induced cell death, whereas it completely blocked ABT-737/etoposide-induced apoptosis used as a positive control for caspase-dependent cell death (Fig. 3a). Consistently, no caspase-3 activation was detected upon treatment with loperamide, pimozide, STF-62247 or IM/TIC in contrast to staurosporine (STS) as a positive control (Fig. 3b), indicating that loperamide, pimozide, STF-62247 and IM/TIC induced cell death largely in a caspase-independent manner. In addition, the ferroptosis inhibitor ferrostatin-1 (Fer-1) failed to block cell death by loperamide, pimozide, STF-62247, or IM/TIC, whereas it efficiently blocked cell death induced by the GPX4 inhibitor RSL3 (Fig. 3c) that was used as a positive control for ferroptosis^32^. Similarly, addition of the receptor-interacting protein kinase (RIPK)1 inhibitor necrostatin-1s (Nec-1s) failed to block cell death induced by loperamide, pimozide, STF-62247 or IM/TIC, whereas Nec-1s profoundly protected HT-29 colon carcinoma cells from cell death induced by a combination of tumor necrosis factor (TNF)α, the Smac mimetic BV6 and zVAD.fmk (Fig. 3d), a well-described model of necroptosis^33^. Taken together, these findings indicate that apoptosis, ferroptosis and necroptosis are not the main execution pathways during loperamide-, pimozide- and STF-62247-induced cell death.

### Loperamide, pimozide, and STF-62247 induce dephosphorylation of mTORC1 and S6K

**Fig. 4:**
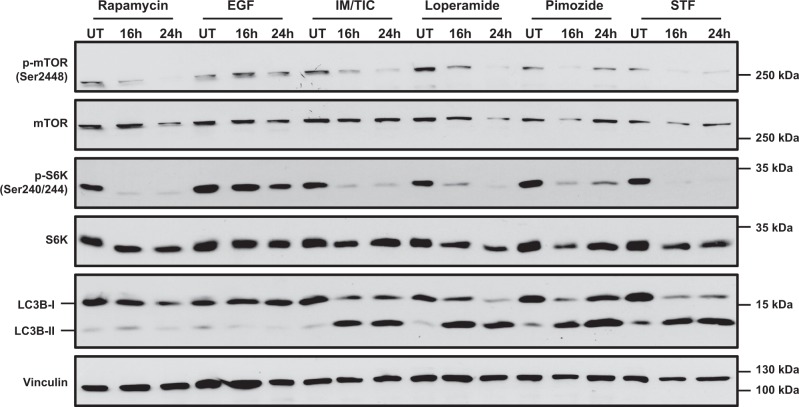
Loperamide, pimozide, and STF-62247 induce dephosphorylation of mTOR and S6K. MZ-54 WT cells were treated with 100 nM rapamycin, 100 ng/mL EGF, 20 µM IM/100 µM TIC, 17.5 µM loperamide, 15 µM pimozide, or 40 µM STF-62247 for the indicated time points followed by Western blotting with vinculin as loading control. UT untreated, EGF epidermal growth factor, IM imipramine hydrochloride, TIC ticlopidine, LOP loperamide, PIMO pimozide, STF STF-62247

mTORC1 is a well-described negative regulator of autophagy^34^. It inhibits this pathway by phosphorylating few ATG proteins^35^. mTORC1 itself can be activated through phosphorylation at Ser2446 by protein kinase B (PKB), which leads to inhibition of autophagy^36,37^. Since this phosphorylation site acts as a switch controlling the activity and function of mTORC1, we investigated whether loperamide, pimozide, and STF-62247 induce dephosphorylation of mTORC1 at Ser2446, as this would allow for activation of autophagy^37^. Indeed, loperamide, pimozide, STF-62247, and IM/TIC markedly reduced phosphorylation of mTORC1, similar to the well-described autophagy inducer rapamycin^38^ (Fig. 4). In addition, we assessed the phosphorylation status of ribosomal protein S6 kinase I (S6K), one of the downstream targets of mTORC1^39^. In line with the observed dephosphorylation of mTORC1, all compounds caused dephosphorylation of S6K (Fig. 4). Since rapamycin has been reported to induce autophagy by preventing phosphorylation of mTORC1 at Ser2446^34^, we tested whether this drug could also induce autophagy-dependent cell death. Rapamycin, however, did not induce cell death in MZ-54 cells (Suppl. Fig. S4 A-C) at a concentration that inhibited phosphorylation of mTORC1 and S6K (Fig. 4). Together, this set of experiments indicates that loperamide, pimozide, and STF-62247 negatively regulate mTORC1, which in turn may lead to increased autophagy.

In addition to mTORC1, also ROS are well-known upstream modulators of autophagy^40^. To test whether ROS formation contributes to loperamide, pimozide- and STF-62247-induced ACD we investigated the effect of different ROS scavengers, i.e. the water-soluble vitamin E-derivate trolox, the lipid-soluble vitamin E-derivate α-tocopherol (α-Toc) and reduced glutathione (GSH)^41,42^. The ROS-inducing compound RSL3 was used as a positive control for ROS-induced cell death^43^. Preincubation with α-Toc significantly rescued loperamide- and pimozide-induced cell death of MZ-54 WT and *ATG7* KO cells, while trolox and GSH had no effect (Suppl. Fig. S5 A). Consistently, loperamide and pimozide markedly triggered ROS production, while STF-62247 slightly increased ROS levels in WT and *ATG7* KO cells (Suppl. Fig. S5 B). These findings indicate that loperamide- and pimozide-induced ACD is associated with ROS formation.

### Loperamide, pimozide, and STF-62247 induce robust hallmarks of autophagy in GBM cells

**Fig. 5:**
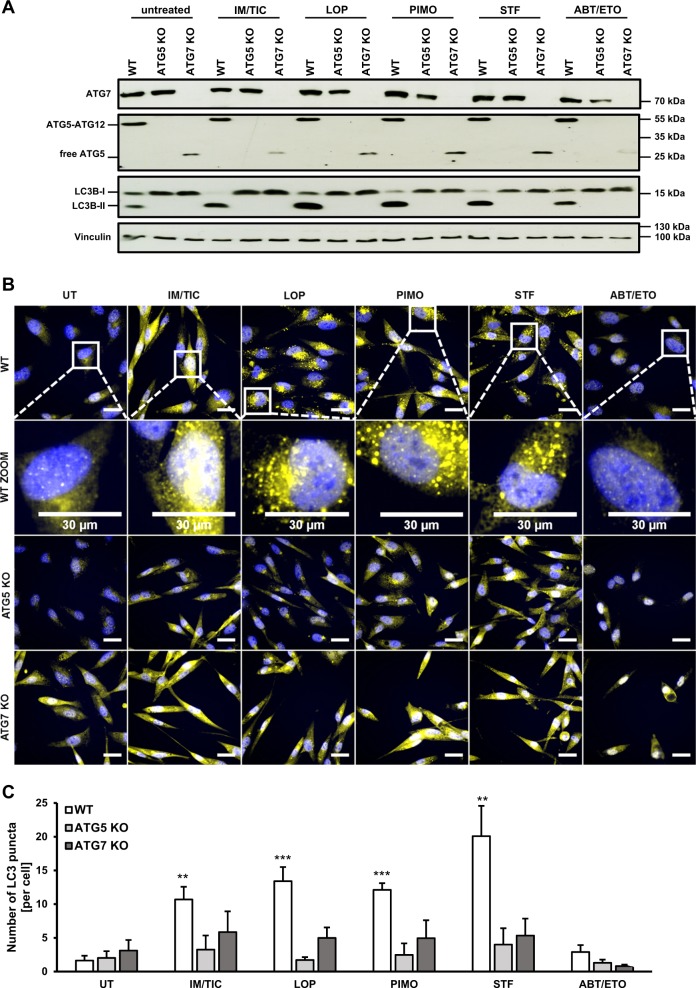
Loperamide, pimozide, and STF-62247 induce robust hallmarks of autophagy in GBM cells. **a** MZ-54 cells were treated with 20 µM IM/100 µM TIC, 17.5 µM loperamide, 15 µM pimozide, 40 µM STF-62247, and 25 µM ABT-737/50 µM etoposide for 24 h followed by detection of vinculin, ATG7, ATG5, and LC3B protein levels by Western blotting with vinculin as loading control. **b** MZ-54 cells were treated as indicated in **a** for 24 h and the formation of LC3B puncta was imaged using anti-LC3B immunofluorescence staining. Representative images over 25 microscopic views per sample are shown. **c** Quantification of mean LC3B puncta per cell upon the indicated treatment. Mean and SEM of 3−6 independent experiments performed for 25 sites per sample are shown. Scale bar = 30 µm. Significances after drug treatment of WT, *ATG5*, and *ATG7* KO cells are calculated versus untreated cells of the corresponding cell line. **p < 0.01, ***p < 0.001. UT untreated, IM imipramine hydrochloride, TIC ticlopidine, LOP loperamide, PIMO pimozide, STF STF-62247, ABT ABT-737, ETO etoposide

To confirm that loperamide, pimozide, and STF-62247 indeed trigger autophagy in MZ-54 cells, we initially assessed LC3B lipidation as a well-characterized marker for autophagy^44^. Indeed, all three compounds as well as the combination of IM and TIC induced a strong increase in lipidated LC3B-II levels compared to untreated cells or cells treated with the apoptosis stimulus ABT-737/etoposide that was used as a negative control (Fig. 5a). Kinetic analysis revealed that LC3B lipidation upon treatment with loperamide, pimozide, STF-62247, and IM/TIC occurred in a time-dependent manner (Suppl. Fig. S6). Strong induction of autophagy occurred 3−6 h after the addition of loperamide, pimozide, and STF-62247 as well as 24 h after adding IM/TIC (Suppl. Fig. S6). Moreover, we confirmed that loperamide, pimozide, and STF-62247 enhanced LC3B lipidation in LN-229 and U343 cells as well (Suppl. Fig. S7).

Upon induction of autophagy, LC3 and GABARAP family proteins associated with expanding phagophores and autophagosomes appear as distinct puncta-like cytoplasmic accumulations which can be assessed by immunofluorescence^12,45^. Therefore, we monitored the induction of autophagy by immunofluorescent staining of endogenous LC3B puncta. Notably, treatment of WT MZ-54 cells with loperamide, pimozide, STF-62247 or IM/TIC stimulated a strong accumulation of LC3B compared to a diffuse staining pattern of LC3B in untreated control cells (Fig. 5b). ABT-737/etoposide treatment was used as a negative control (Fig. 5b). Quantification revealed a significant increase in LC3B puncta upon treatment with loperamide, pimozide, STF-62247 or IM/TIC compared to untreated control cells (Fig. 5c). In contrast, LC3B punctate formation was almost completely blocked in *ATG5* or *ATG7* KO MZ-54 cells, as expected (Fig. 5c). This set of experiments strongly suggests that loperamide, pimozide, and STF-62247 trigger canonical autophagy in GBM cells.

### Loperamide and pimozide induce ultrastructural hallmarks of autophagy in GBM cells

**Fig. 6:**
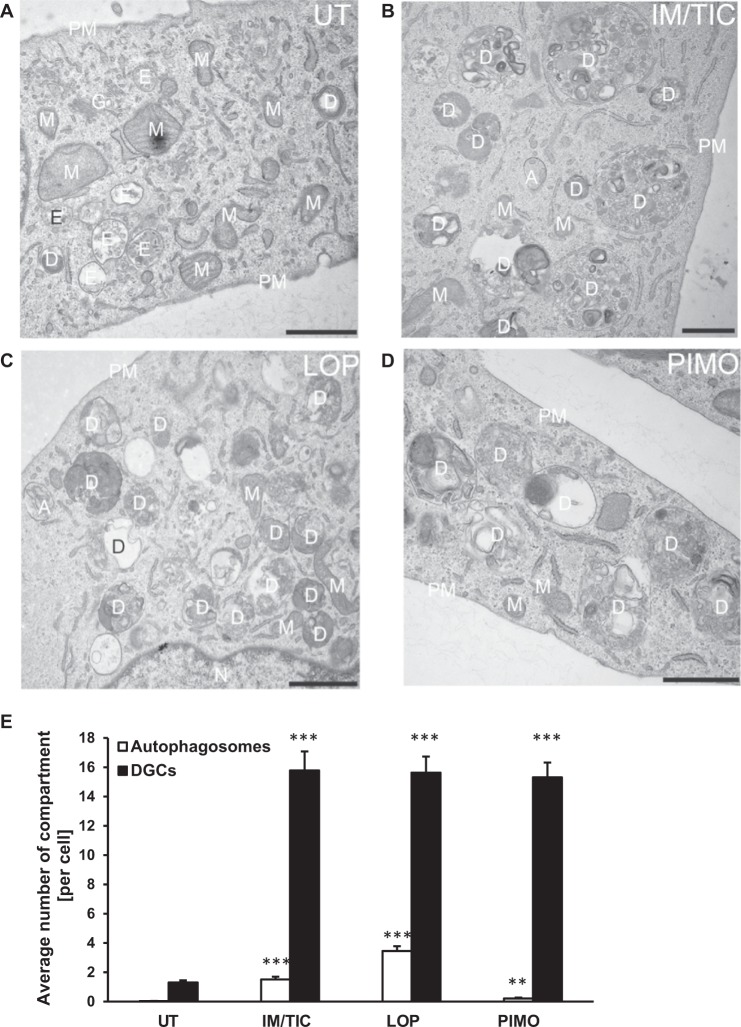
Loperamide and pimozide induce ultrastructural hallmarks of autophagy in GBM cells. **a–d** MZ-54 WT cells were left untreated (**a**, UT) or treated with 20 µM IM/100 µM TIC (**b**, IM/TIC), 17.5 µM loperamide (**c**, LOP) or 15 µM pimozide (**d**, PIMO) for 48 h before being processed for electron microscopy. A, autophagosome; D, degradative compartment; E, endosome (early or late); G, Golgi apparatus; M, mitochondria; N, nucleus; PM, plasma membrane. Scale bar = 1 µm. **e** The average number of autophagosomes and degradative compartments per cell section was determined as described in Materials and Methods. Significances are calculated versus untreated cells. **p < 0.01, ***p < 0.001. UT untreated, IM imipramine hydrochloride, TIC ticlopidine, LOP loperamide, PIMO pimozide

To further characterize autophagic changes upon exposure to IM/TIC, loperamide or pimozide, we performed a detailed ultrastructural analysis using electron microscopy. In contrast to the untreated control, treatment with IM/TIC, loperamide or pimozide induced massive formation of heteromorphous degradative compartments (DGC), which include lysosomes, amphisomes (i.e. autophagosomes fused with endosomes) and autolysosomes (Fig. 6a–e). In most cases, these DGC contained electron-dense material that likely reflects the presence of cytoplasmic material being degraded. In addition, we also observed a significant increase in autophagosomes per cell section upon treatment with IM/TIC, loperamide and pimozide (Fig. 6b, c, e). Notably, the increase of DGC was shown to be more pronounced than the increase in autophagosomes, pointing to a rapid fusion of autophagosomes with lysosomes that is indicative of an enhanced autophagic flux. Together, this ultrastructural analysis highlights that treatment with IM/TIC, loperamide and pimozide triggers morphological hallmarks of autophagy.

### Loperamide, pimozide and STF-62247 enhance the autophagic flux in GBM cells

**Fig. 7:**
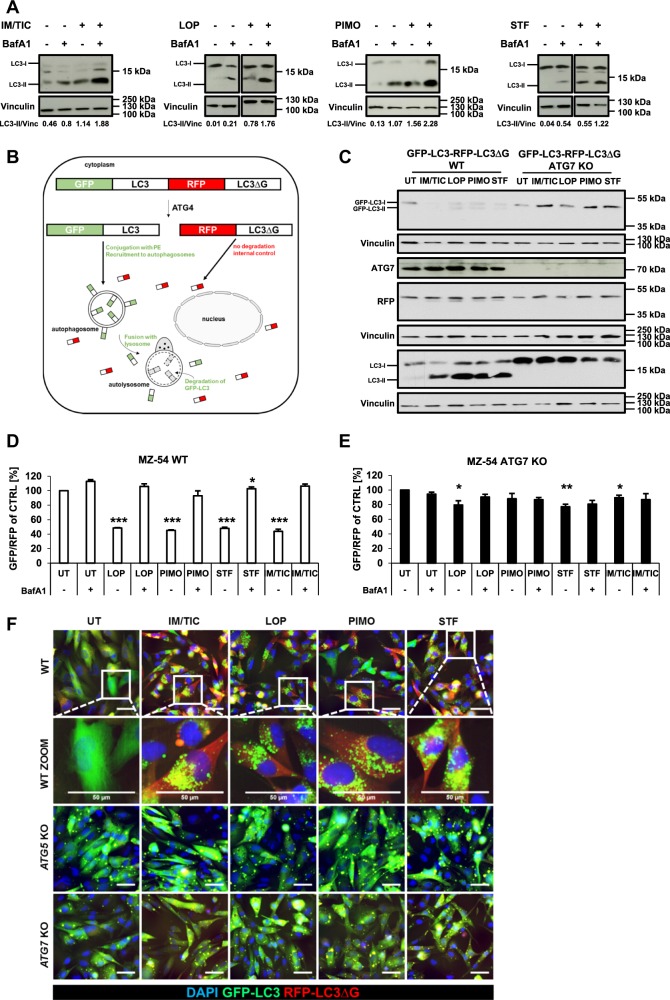
Loperamide, pimozide, and STF-62247 enhance the autophagic flux in MZ-54 cells. **a** MZ-54 cells were treated with 20 µM IM/100 µM TIC, 17.5 µM loperamide, 15 µM pimozide, and 40 µM STF-62247 for 8, 2, 4 and 3 h, respectively. BafA1 was added 4 h before cell harvesting at a final concentration of 40 nM. Western blotting was performed with the indicated antibodies and vinculin as loading control. For quantification, LC3-II band intensities were normalized to vinculin band intensities. **b** Schematic representation of the GFP-LC3B-RFP-LC3BΔG autophagy flux sensor. Upon expression, the GFP-LC3B-RFP-LC3BΔG fusion protein is cleaved by the ATG4 proteases after which GFP-LC3B becomes conjugated to PE and localizes to autophagosomes which eventually fuse with lysosomes, inducing degradation of GFP-LC3B. RFP-LC3BΔG remains in the cytosol, where it serves as internal control. Scheme adapted from Kaizuka et al.^48^ **c** Stable GFP-LC3B-RFP-LC3BΔG-expressing MZ-54 WT or *ATG7* KO cells were treated as indicated in **a** for 16 h followed by Western blotting with vinculin as loading control. **d**, **e** Stable GFP-LC3B-RFP-LC3BΔG-expressing MZ-54 WT (**d**) or *ATG7* KO cells (**e**) were treated with 20 µM IM/100 µM TIC, 15 µM loperamide, 15 µM pimozide or 40 µM STF-62247 for the indicated time points followed by flow cytometry. BafA1 was added 4 h before the measurement at a final concentration of 40 nM. Mean and SEM of three independent experiments performed in triplicate are shown. **f** Fluorescence microscopy of stable GFP-LC3B-RFP-LC3BΔG-expressing MZ-54 WT, *ATG5* KO and *ATG7* KO cells was performed after 16 h of treatment with 20 µM IM and 100 µM TIC, 17.5 µM loperamide, 15 µM pimozide, and 40 µM STF-62247. Scale bar = 50 µm. Significances are calculated versus untreated cells of the same cell line. *p < 0.05, **p < 0.01, ***p < 0.001. UT untreated, IM imipramine hydrochloride, TIC ticlopidine, LOP loperamide, PIMO pimozide, STF STF-62247

An increase in autophagosomes can be related either to an increased autophagic flux or to a block of the autophagosomal-lysosomal fusion^46^. To address this point we treated MZ-54 cells with loperamide, pimozide, STF-62247 and IM/TIC in the absence and presence of bafilomycin A1 (BafA1). Since BafA1 inhibits the acidification of lysosomes and thus the degradation of the autophagosomal cargoes including the members of the LC3 protein family, enhanced LC3B-II levels upon treatment with BafA1 reflect enhanced stimulation of the autophagic flux^47^. Notably, treatment with loperamide, pimozide, STF-62247 or IM/TIC caused enhanced LC3B lipidation upon addition of BafA1 compared to treatment in the absence of BafA1 (Fig. 7a), pointing to an increase in the autophagic flux.

To accurately corroborate the effects of these compounds on the autophagic flux we used the dual GFP/RFP-LC3B autophagy flux sensor system that has been described recently^48^. This system is based on the expression of the GFP-LC3B-RFP-LC3BΔG fusion protein, composed of green fluorescent protein (GFP) fused to WT LC3B and red fluorescent protein (RFP) fused to the LC3B Gly120 mutant (Fig. 7b). Upon ectopic expression, this fusion protein is intracellularly cleaved by the ATG4 proteases into equimolar ratios (Fig. 7b). GFP-LC3B localizes to autophagosomes and eventually becomes partly degraded in autolysosomes upon induction of autophagy. However, the mutated RFP-LC3BΔG cannot be conjugated to autophagosomes and remains cytosolic without being turned over by autophagy, thus serving as internal control (Fig. 7b). We therefore assessed GFP and RFP protein levels by Western blotting upon the induction of autophagy by loperamide, pimozide, and STF-62247. Importantly, all three compounds led to a decrease of GFP protein levels, whereas RFP levels remained stable (Fig. 7c). In contrast, both GFP and RFP levels remained stable upon compound treatment of *ATG7* KO cells (Fig. 7c).

To confirm these findings we quantified GFP/RFP ratios by flow cytometry. This analysis showed that loperamide, pimozide, STF-62247, and IM/TIC did indeed reduce the GFP/RFP fluorescence ratio in WT MZ-54 cells, reflecting an enhancement of the autophagic flux (Fig. 7d). Consistently, the addition of BafA1 prevented the decrease of the GFP/RFP ratio by preventing lysosomal degradation of GFP-LC3B (Fig. 7d). In contrast, *ATG7* KO MZ-54 cells expressing the GFP/RFP LC3B sensor did not display differences in GFP/RFP ratios upon treatment with any of the compounds (Fig. 7e).

Next, we validated these findings by fluorescence microscopy. Indeed, treatment of WT GFP/RFP LC3B reporter-expressing cells with loperamide, pimozide, STF-62247, and IM/TIC led to a strong accumulation of distinct cytoplasmic GFP-positive puncta, likely representing expanding phagophores or autophagosomes, and an overall visible decrease of GFP fluorescence, whereas RFP fluorescence remained stable (Fig. 7f).

In addition, fluorescence microscopy was performed on MZ-54 cells stably expressing LC3B fused to mRFP and GFP. Upon induction of autophagy, this mRFP-GFP-LC3B fusion protein localizes to autophagosomes and their precursor structures and emits yellow fluorescence^49^. As soon as this fusion protein reaches the lysosomal lumen, the GFP fluorescence becomes quenched due to the acidic environment, leading to a net stabilization of red fluorescence signals^49^. Therefore, accumulation of red fluorescent signals indicates an induction of the autophagic flux^49^. Indeed, treatment with loperamide, pimozide, STF-62247, or IM/TIC led to a marked accumulation of yellow and red puncta, representing autophagosomes and autolysosomes, respectively (Suppl. Fig. S8 A-B). As expected, addition of BafA1 inhibited the formation of autolysosomes (Suppl. Fig. S8 A-B). Together, these findings confirm that loperamide, pimozide, and STF-62247 induce autophagy-dependent cell death by enhancing the autophagic flux.

## Discussion

As ACD has been described as a possible therapeutic approach in GBM, the identification of agents that trigger ACD has attracted considerable interest^6,24,26,29,50,51^. Using CRISPR/Cas9-derived ATG5 and ATG7-deficient models, we identified loperamide, pimozide, and STF-6224 as three novel candidates that induce biochemical and cellular hallmarks of autophagy and autophagy-dependent cell death in several GBM cell lines.

Several lines of evidence confirm the induction of autophagy and subsequent cell death by these compounds. First, biochemical and cellular characteristics of autophagy as well as cell death induced by loperamide, pimozide or STF-6224 were significantly reduced by depletion of ATG5 or ATG7 expression. Second, we demonstrated that loperamide, pimozide, and STF-62247 induced an increase in the autophagic flux of MZ-54 cells that was potentiated by inhibition of lysosomal maturation and reduced by loss of ATG5 or ATG7 expression. Third, loperamide- and pimozide-treated MZ-54 cells were characterized by distinct and prominent ultrastructural hallmarks of autophagy, which are generally considered as a gold standard for the analysis of autophagy^23^. Fourth, we demonstrated that cell death induced by loperamide, pimozide, and STF-62247 was not primarily mediated via apoptosis, necroptosis or ferroptosis, as typical pharmacological inhibitors of these cell death modalities largely failed to prevent cell death. Therefore, cell death induced by loperamide, pimozide, and STF-62247 is dependent on autophagy and can be classified as ACD^17,23,52^.

STF-62247 has previously been discovered in a small molecule-based screen to induce ACD in renal carcinoma cells^53^. By performing a screen in a yeast KO collection, a network of vesicular trafficking between the endoplasmic reticulum (ER), the trans-Golgi network and lysosomes was suggested as a target of STF-62247^53^. Consistent with this hypothesis, several studies highlighted the relevance of the trans-Golgi network for autophagosome formation and the initiation of autophagy^54,55^.

While both loperamide and pimozide have previously been reported to stimulate autophagy in a high-throughput fluorescence microscopy-based screen of H4 GBM cells^56^, our study is the first to show that loperamide and pimozide trigger ACD. Loperamide is a Food and Drug Administration (FDA)-approved piperidine derivate that inhibits voltage-gated L-type calcium (Ca^2+^) channels^57^. Pimozide is an FDA-approved diphenylbutylpiperidine that targets D2 dopaminergic receptors^58^. It is used in the clinic for the treatment of schizophrenia, but also as an experimental anticancer drug^59,60^. Interestingly, it has been demonstrated that pimozide is a potent inhibitor of low voltage-gated T-type Ca^2+^ channels^61^.

In several studies, Ca^2+^ channel antagonists have been associated with autophagy regulation; however, their exact effects on autophagy are still being controversially discussed^62^. For instance, an increase in intracellular Ca^2+^ has been shown to inhibit mTORC1 signaling through Ca^2+^/calmodulin-dependent protein kinase 2 (CAMKK2/CaMKKβ) and AMP-activated protein kinase (AMPK), leading to accumulation of autophagosomes^63^. On the other hand, an inhibitory role of enhanced intracellular Ca^2+^ levels on autophagy has been reported as well. Increases in Ca^2+^ activate calpains and adenylate cyclase, leading to increased levels of 3′-5′-cyclic adenosine monophosphate (cAMP)^64^. cAMP stimulates inositol triphosphate (IP3) production that activates inositol 1,4,5-triphosphate receptors (ITPRs) on the ER membranes to secrete Ca^2+^, resulting in autophagy inhibition by maintaining enhanced mTORC1 activity^65^. Moreover, Ca^2+^ release from the ER and the subsequent decrease in intra-ER Ca^2+^ levels were shown to promote misfolding of the lysosomal proton pump vATPase, leading to impaired lysosomal acidification and autophagy^66,67^. According to this scenario, inhibition of Ca^2+^ channels through loperamide and pimozide may enhance autophagy indirectly through a release from autophagy inhibition. Interestingly, apart from enhancing autophagy through lowering IP3 levels, inhibition of voltage-gated channels has also been shown to induce autophagy through inhibition of calpain-mediated cleavage of ATG5^68^.

Moreover, our study suggests that loperamide, pimozide, and STF-62247 regulate autophagy through dephosphorylation of mTORC1, a master regulator of autophagy^69^. This is consistent with recent findings showing that STF-62247 inhibits mTORC1^70^. Loperamide and pimozide may lead to a decrease in intracellular Ca^2+^ levels through inhibition of voltage-gated Ca^2+^ channels. Interestingly, a rise of intracellular Ca^2+^ has been reported to promote binding of Ca^2+^-bound calmodulin (CALM) to PIK3C3/VPS34, resulting in mTORC activation and autophagy suppression^71^. In line with these findings, we observed that loperamide- and pimozide-induced autophagy is accompanied by dephosphorylation and inactivation of mTORC1. Furthermore, several studies highlighted a role for ROS in autophagy induction^15,40^. For instance, it was shown that ROS formation can contribute to ACD while antioxidants can reverse autophagy, suggesting that ROS formation precedes autophagy under certain circumstances^40,72^.

So far, there have been few studies on selective mediators of ACD. Recently, glucocerebrosidase (GBA1) has been identified by a signalome-wide shRNA-based cell viability screen as a critical mediator of autophagic self-consumption and ACD^25^. Yu et al. reported that autophagy promotes cell death by selective digestion of the ROS scavenger catalase^21^. A study by Karch et al. demonstrated that the BCL-2 family members BAX and BAK are essential for serum starvation-induced ACD in mouse embryonic fibroblasts (MEFs) by increasing lysosomal membrane permeability^73^. Moreover, TP53 has previously been shown to regulate sphingosine kinase 1 (SPHK1)-induced ACD in colon carcinoma cells^74^.

In recent years, several scenarios have been developed to explain how increased autophagy can lead to cell death. The simplest explanation is a threshold effect of autophagy: in this model, extensive and prolonged hyperactivation of autophagy triggers cellular self-digestion via the autophagosomal-lysosomal pathway beyond the point that allows cell survival^18,19,75^. Indeed, our present study shows that loperamide, pimozide, and STF-62247 induced a strong accumulation of LC3B-positive autophagosomes and autolysosomes prior to cell death over a period of 48 h, hence supporting the hypothesis of a threshold effect which possibly turns autophagy into a detrimental process. However, it remains subject to future investigations to identify the pathways and factors that are responsible for ACD upon treatment with loperamide, pimozide and STF-62247.

An important prerequisite for the delivery of compounds to brain tumors is their transfer through the blood−brain barrier, which tightly regulates the passage of soluble molecules from the blood to the brain^76^. Pimozide is used in the clinic for treatment of schizophrenia^59^, loperamide was shown to be delivered to the brain when loaded to polysorbate 80-coated poly(butyl cyanoacrylate) nanoparticles^77^ and STF-62247 may well pass the blood−brain barrier due to its hydrophobic nature and small size. This suggests that all three compounds may be able to reach the brain compartment.

In summary, we have identified in the present study that loperamide, pimozide, and STF-62247 induce ACD in GBM cells. Essentially, until now two of these compounds, i.e. loperamide and pimozide, have not been reported to induce ACD in any cellular model system. Thus, our study emphasizes the critical role of autophagy and ACD in GBM cells and provides novel options for the treatment of these apoptosis-resistant tumors.

## Materials and methods

### Cell lines and chemicals

The human glioma cell lines MZ-54^26,29^, LN-229, and U343 GOS-3 as well as the human colon carcinoma cell line HT-29 were cultured in DMEM GlutaMAX medium (Life Technologies, Inc., Eggenstein, Germany) supplemented with 10% fetal calf serum (FCS) (Life Technologies, Inc., Eggenstein, Germany) and 1% penicillin/streptomycin (Life Technologies, Inc., Eggenstein, Germany) at 37 °C and 5% CO_2_. Cells were regularly tested for mycoplasma infection. Cells were authenticated by STR profiling at DSMZ (Sammlung von Mikroorganismen und Zellkulturen GmbH). Imipramine hydrochloride, ticlopidine, Fer-1, pimozide, epidermal growth factor (EGF), α-Toc, (±)-6-Hydroxy-2,5,7,8-tetramethylchromane-2-carboxylic acid (trolox) and puromycin were purchased from Sigma-Aldrich (St. Louis, Missouri, USA). Loperamide hydrochloride, rapamycin and STS were purchased from Enzo Life Sciences (Lausen, Switzerland). STF-62247 was purchased from Santa Cruz Biotechnology, Inc. (Dallas, Texas, USA), RSL3 from InterBioScreen (InterBioScreen ltd., Russia), etoposide from TEVA GmbH (Ulm, Germany), ABT-737 from Selleck Chemicals (Houston, Texas, USA) and G418 and reduced GSH from Carl Roth (Karlsruhe, Germany). The caspase inhibitor zVAD.fmk was purchased from Bachem (Heidelberg, Germany) and Nec-1s from Biomol (Hamburg, Germany). The Smac mimetic BV6 was kindly provided by Genentech Inc. (South San Francisco, CA, USA). Recombinant human TNFα was purchased from Biochrom (Berlin, Germany).

### Generation of *ATG5/7* CRISPR/Cas9 KO cells

To generate *ATG5* and *ATG7* KO cells, guide RNAs for *ATG5* (MZ-54 *ATG5* KO: TCAGGATGAGATAACTGAAA and CCTCTAATGCTACCACTCAG, U343 *ATG5* KO: AAGATGTGCTTCGAGATGTG and CCTCTAATGCTACCACTCAG) and *ATG7* (MZ-54 *ATG7* KO: AATAATGGCGGCAGCTACGG and AAAGCTGACACTATACTGG, LN-229 *ATG7* KO: AATAATGGCGGCAGCTACGG and AAGCTGACACTATACTGG) were cloned into SpCas9(BB)-2A-GFP (PX458) or pSpCas9(BB)-2A-Puro (PX459) V2.0 from Feng Zhang (Addgene plasmids #48138 and #62988, respectively) by using *Bbs*I according to standard cloning procedures^78^. Plasmids were verified using DNA sequencing. Lipofectamine 3000 was used to transfect both ATG5 and ATG7 sgRNAs into the corresponding GBM cells (DNA/Lipofectamine 3000 ratio 1:1.5) according to the manufacturer’s instructions. 72 h after transfection, SpCas9(BB)-2A-GFP-transfected cells were sorted into a 24-well plate with an FACS Aria II cell sorter (BD Biosciences). 48 h after transfection, cells transfected with pSpCas9(BB)-2A-Puro were selected with 1 µg/mL puromycin. Next, single cell dilution into 96-well plates was performed using conditioned medium containing 50% sterile-filtered medium from cultured cells and 50% fresh medium for 72 h in order to ensure growth under single cell conditions. *ATG5* and *ATG7* KO colonies were expanded and confirmed by PCR analysis and western blot.

### Screening of autophagy-inducing compounds

Parental MZ-54 cells as well as *ATG5* KO cells were seeded at 6500 cells/96-well followed by treatment with autophagy-inducing compounds of the Enzo Screen-Well™ library (Enzo Life Sciences, Lausen, Switzerland). Compounds were added to final concentrations between 100 nM and 100 µM. Cell death was assessed after 48 h by fluorescence-based microscope analysis of propidium iodide (PI) uptake using Hoechst 33342 and PI double staining (Sigma-Aldrich, St. Louis, Missouri, USA) as well as ImageXpress Micro XLS Widefield High-Content Analysis System and MetaXpress Software according to the manufacturer’s instructions (Molecular Devices Sunnyvale, CA, USA).

### Generation of pMRX-IP-GFP-LC3B-RFP-LC3BΔG-expressing cells and determination of autophagic flux

Parental MZ-54 and ATG5/7 KO cells were transfected with pMRX-IP-GFP-LC3B-RFP-LC3B∆G (Addgene plasmid # 84572, a gift from Noboru Mizushima^48^) by using Lipofectamine 2000 according to the manufacturers’ instructions. 48 h after transfection, cells were selected with 1 µg/mL puromycin for 7 days. For determination of autophagic flux, cells were seeded on Greiner black micro-clear 96-well plates at 10,000 cells/well and imaged with the ImageXpress Micro XLS Widefield High-Content fluorescence microscope (Molecular Devices Sunnyvale, CA, USA) by using the 60× objective and the FITC and Texas Red filter system for imaging of GFP-LC3B and RFP-LC3B∆G, respectively. Image analysis was performed with ImageJ (v1.51t).

### Generation of mRFP-GFP-LC3B-expressing cells and measurement of the autophagic flux

Parental MZ-54 and MZ-54 *ATG5/7* KO cells were transfected with the mRFP-GFP-LC3B plasmid (Addgene #21074) by using Lipofectamine 3000 according to the manufacturer’s instructions. 48 h after transfection, cells were selected with 1 mg/mL G418. For determination of the autophagic flux, cells were seeded into chamber slides without selection of antibiotic at 12,000 cells/well, treatment was performed as indicated and cells were fixed with 4% paraformaldehyde for 10 min followed by ice-cold methanol for 5 min. After washing with 0.1% triton-X in phosphate-buffered saline (PBS), cover glasses were fixed with mounting medium containing DAPI (Dianova, Hamburg, Germany). Microscope images were taken with the Nikon Eclipse TE2000-S microscope and NIS Elements AR 3.2 software (Nikon Instruments Europe BV, Amsterdam, Netherlands) with 60× magnification.

### Assessment of the autophagic flux by flow cytometry

To determine autophagic flux by FACS, cells stably expressing pMRX-IP-GFP-LC3B-RFP-LC3BΔG were pelletized and resuspended in 50 µL of PBS. Measurements were performed with an FACS Accuri flow cytometer according to the manufacturer’s instructions (BD Biosciences, Heidelberg, Germany). For calculation of GFP/RFP ratios, the mean fluorescence intensity (MFI) ratio of GFP and RFP of untreated WT and ATG7 KO cells was set to 100%. MFI ratios of compound-treated samples were then normalized to the corresponding control cell line.

### Determination of cell death

Cell death was measured by fluorescence-based microscope analysis of PI uptake using Hoechst 33342 and PI double staining (Sigma-Aldrich, St. Louis, Missouri, USA) and the ImageXpress Micro XLS Widefield High-Content Analysis System and MetaXpress Software according to the manufacturer’s instructions (Molecular Devices Sunnyvale, CA, USA).

### Immunofluorescence analyses

For immunofluorescence staining of LC3B, MZ-54 cells were seeded at 10,000 cells/96-well. For immunofluorescence, cells were fixed with 3.7% paraformaldehyde for 10 min, followed by a washing step with PBS and permeabilization with 0.1% Triton-X diluted in PBS for 10 min. After washing with PBS, cells were blocked with an antibody dilution buffer (ADB) containing 0.9% NaCl, 10 mM Tris HCl pH 7.5, 5 mM EDTA and 1 mg/mL BSA for 10 min. Cells were incubated with an antibody against LC3B (Thermo Fisher, PA1-46286) diluted 1:350 in ADB for 1 h. After three washing steps with 0.1% Tween-20 diluted in PBS (PBS-T), cells were incubated with Cy3^TM^ AffiniPure donkey-a-rabbit IgG (Jackson Immuno Research Laboratories, Inc.) diluted 1:800 in ADB for 30 min. After three washing steps with PBS-T, Hoechst 33342 was added to the cells diluted 1:15,000 in PBS followed by image acquisition with the ImageXpress Micro XLS Widefield High-Content Analysis System (Molecular Devices Sunnyvale, CA, USA) by using the 60× objective and the DAPI and TRITC filter system for acquisition of Hoechst-stained nuclei and Cy3^TM^-stained LC3B, respectively. Image analysis was performed using ImageJ 1.51t.

### Caspase activity assay

For measurement of caspase-3-like activity, 30,000 cells were seeded per 24-well. Cells were lysed in lysis buffer containing 10 mM HEPES, pH 7.4, 42 mM KCl, 5 mM MgCl_2_, 1 mM phenylmethylsulfonyl fluoride, 0.1 mM EDTA, 0.1 mM EGTA, 1 mM dithiothreitol (DTT), 1 μg/mL pepstatin A, 1 μg/mL leupeptin, 5 μg/mL aprotinin, 0.5% 3-(3-cholamidopropyldimethylammonio)-1-propane sulfonate (CHAPS). 50 μl of cell lysate were added to 150 µL reaction buffer (25 mM HEPES, 1 mM EDTA, 0.1% CHAPS, 10% sucrose, 3 mM DTT, pH 7.5) containing the fluorigenic substrate Ac-DEVD-AMC (Enzo Life Sciences, Lausen, Switzerland) at a final concentration of 10 μM. 7-Amino-4-methylcoumarin (AMC) fluorescence was monitored for 2 h using a Spark multimode microplate reader (Tecan Group AG, Männedorf, Switzerland). Changes in fluorescence measured over 2 h were normalized to the total protein content of the lysate. Caspase activity was expressed as change in fluorescence units per μg protein and hour.

### Electron microscopy

For conventional transmission electron microscopy, MZ-54 WT cells were treated with the indicated concentrations of the compounds. After 48 h, an equal volume of double strength fixatives (4% paraformaldehyde, 4% glutaraldehyde in 0.1 M cacodylate buffer (pH 7.4)) was added to the cells for 20 min at room temperature, prior to fixing the cells with one volume of 2% paraformaldehyde and 2.5% glutaraldehyde in 0.1 M cacodylate buffer (pH 7.4) for 2 h at room temperature. Cells were then scraped and embedded as previously described^79^. Ultra-thin 70-nm sections were cut using the Leica EM UC7 ultra microtome (Leica Microsystems, Wetzlar, Germany) and stained with uranyl acetate and lead citrate as previously described^79^. Cell sections were analyzed using a CM100bio TEM (FEI, Eindhoven, Netherlands). The average number of autophagosomes and degradative compartments (amphisomes, lysosomes and autolysosomes) per cell section was determined by counting these compartments through 120 cell sections per condition, randomly selected from five independent grids.

### Western blot analysis

Western blot analysis was performed as described previously using RIPA buffer (50 mM Tris-HCl, pH 8, 1% Triton-X, 0.5% sodium deoxycholate, 150 mM sodium chloride and 2 mM magnesium chloride) supplemented with Pierce Nuclease (Thermo Fisher, Waltham, MA, USA)^80^. The following antibodies were used: monoclonal rabbit anti-ATG7, rabbit anti-ATG5, rabbit anti-phospho mTOR (Ser2446), rabbit anti-mTOR, rabbit anti-phospho S6 Ribosomal Protein (Ser240/244), mouse anti-S6 Ribosomal Protein (54D2) (Cell Signaling, Beverly, MA, USA), mouse anti-vinculin (Sigma, Germany) and rabbit anti-LC3B (Thermo Fisher, Waltham, MA, USA). Goat anti-mouse and goat anti-rabbit conjugated to horseradish peroxidase (Santa Cruz Biotechnology, Santa Cruz, CA, USA) as well as enhanced chemiluminescence (Amersham Biosciences, Freiburg, Germany) were used for detection. Representative blots of at least two independent experiments are shown. Quantification of band intensities was performed using ImageJ 1.51t.

### Determination of ROS production

To analyze ROS production, medium was discarded, and cells were stained for 30 min at 37 °C with 5 μM CM-H2DCFDA (Invitrogen). Subsequently, cells were trypsinized and centrifuged for 10 min at 4 °C. Supernatant was discarded and cells were resuspended in phenol red-free RPMI medium (Life Technologies, Inc.) and immediately analyzed by flow cytometry.

### Statistical analysis

Results are expressed as mean ± SEM. Statistical analysis was performed with SigmaPlot (v12.5). Statistical significance of two group data was analyzed by Student’s *t* test (two-tailed). If samples did not pass either the Shapiro−Wilk Normality Test or the Equal Variance test, statistical significance was analyzed by Mann−Whitney Rank Sum Test. *p* values were interpreted as follows: *p < 0.05; **p ≤ 0.01; ***p ≤ 0.001.

## Electronic supplementary material

Supplemental Material
