# Supplementary material for: Loperamide, pimozide, and STF-62247 trigger autophagy-dependent cell death in glioblastoma cells
Source: Cell Death Dis. 2018 Sep 24;9(10):994. doi: 10.1038/s41419-018-1003-1 (PMC6155211; doi:10.1038/s41419-018-1003-1)
Supplement: Supplementary file 1 — Supplemental Material [file 41419_2018_1003_MOESM1_ESM.pdf]

Supplementary Fig. S1

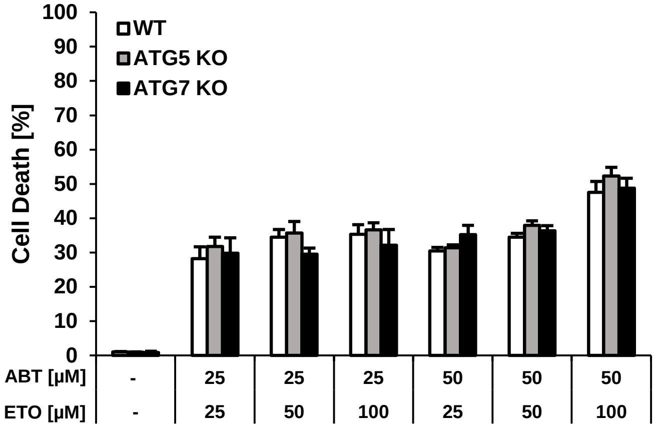

Supplementary Fig. S2

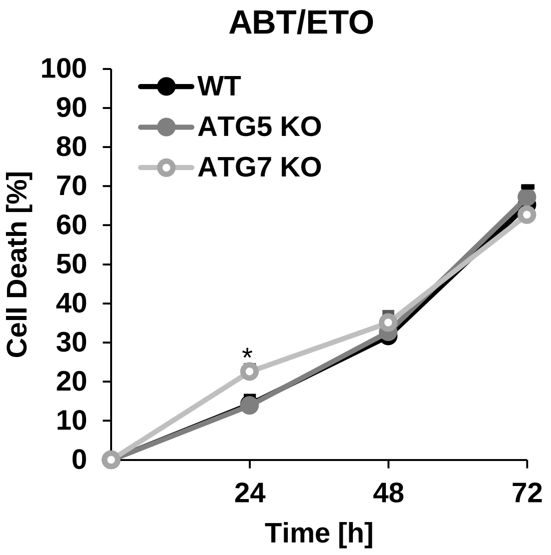

Supplementary Fig. S3

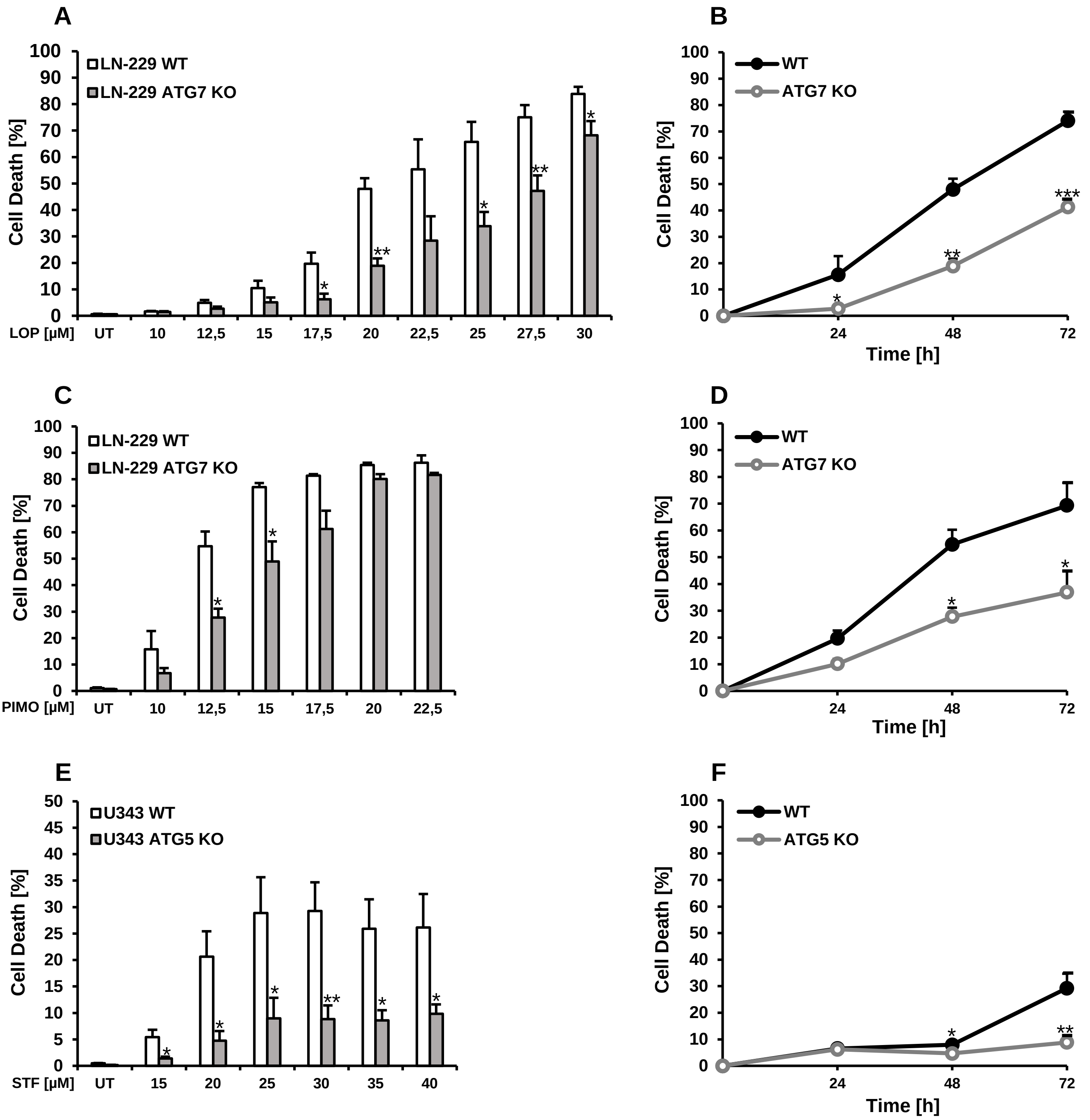

Supplementary Fig. S4

A

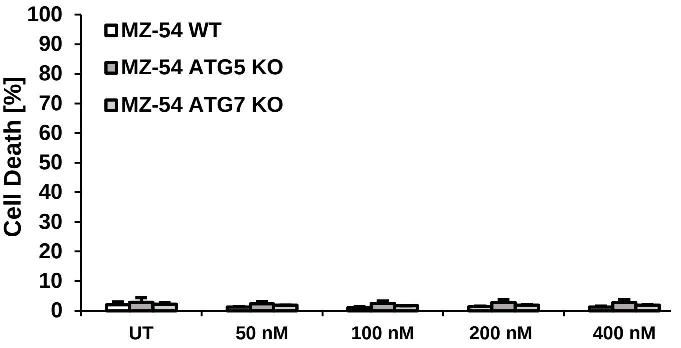

B

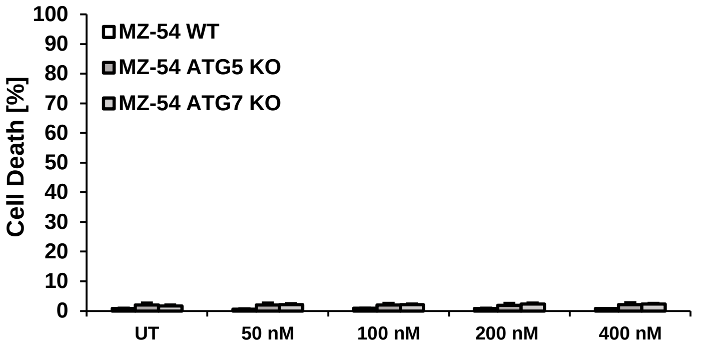

C

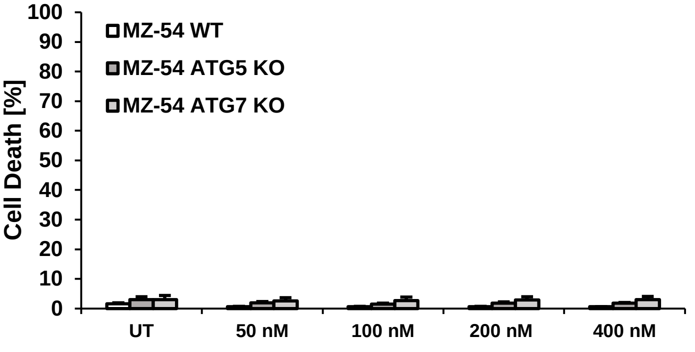

Supplementary Fig. S5

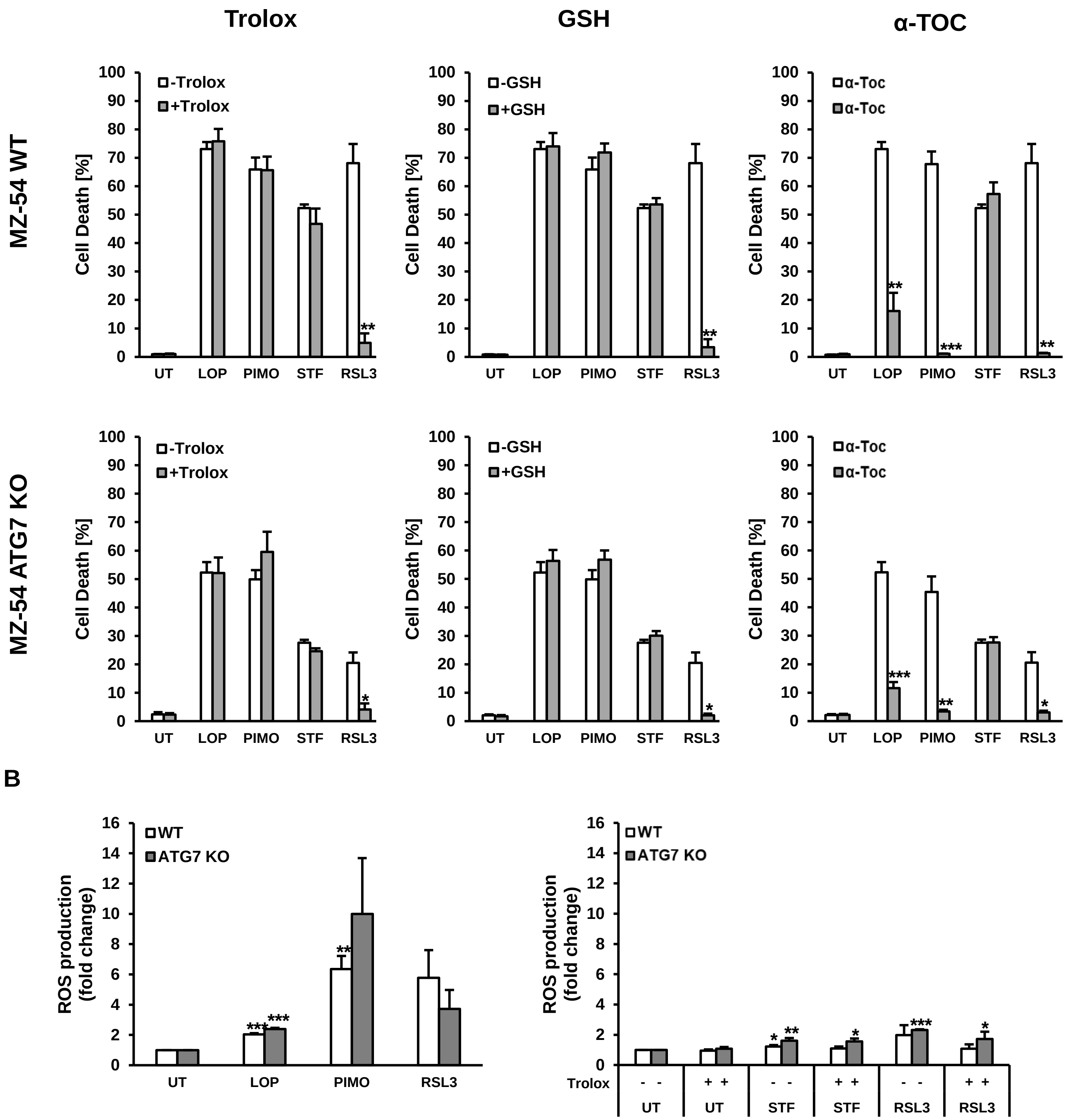

Supplementary Fig. S6

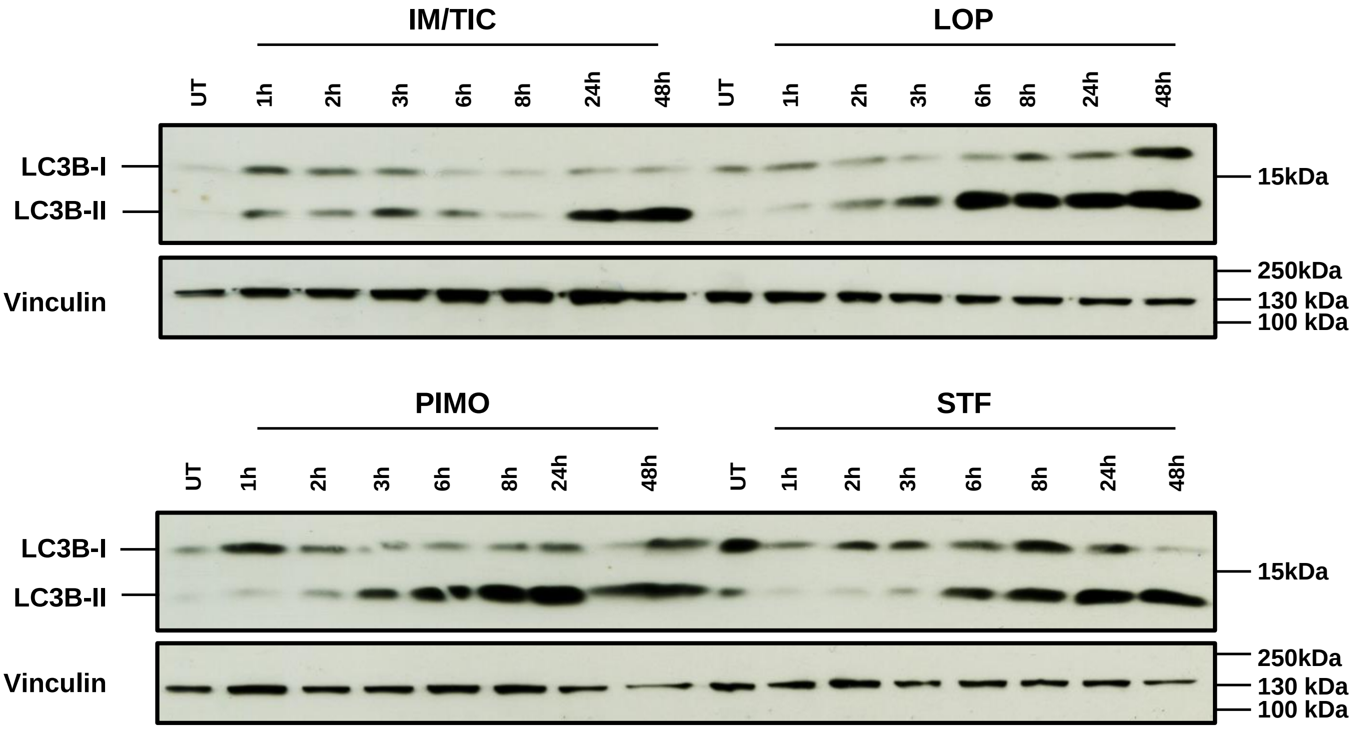

Supplementary Fig. S7

A

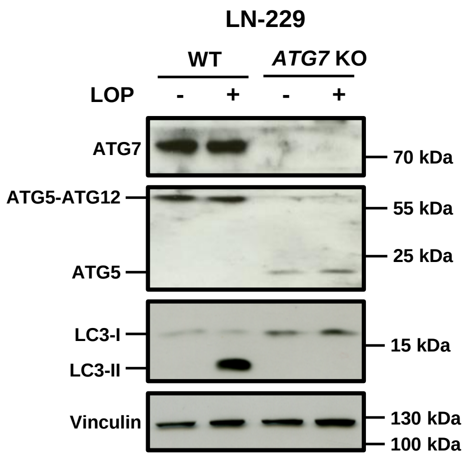

B

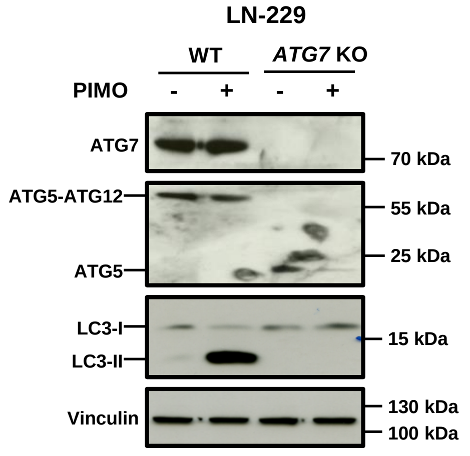

C

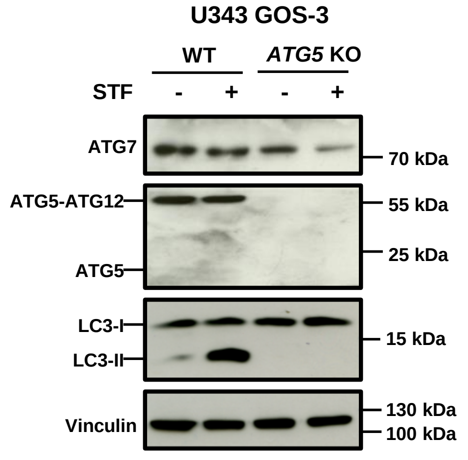

Supplementary Fig. S8

A

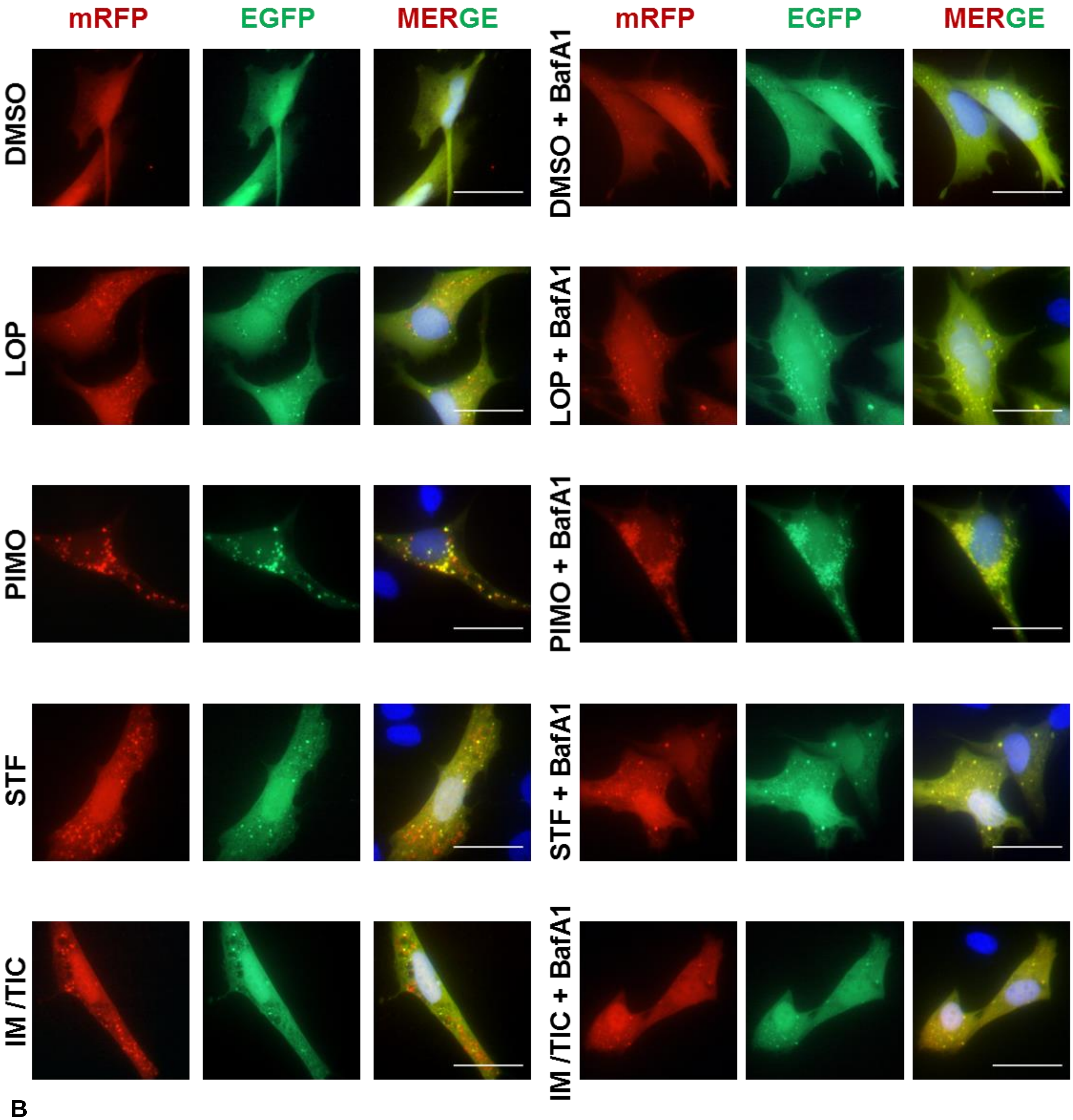

B

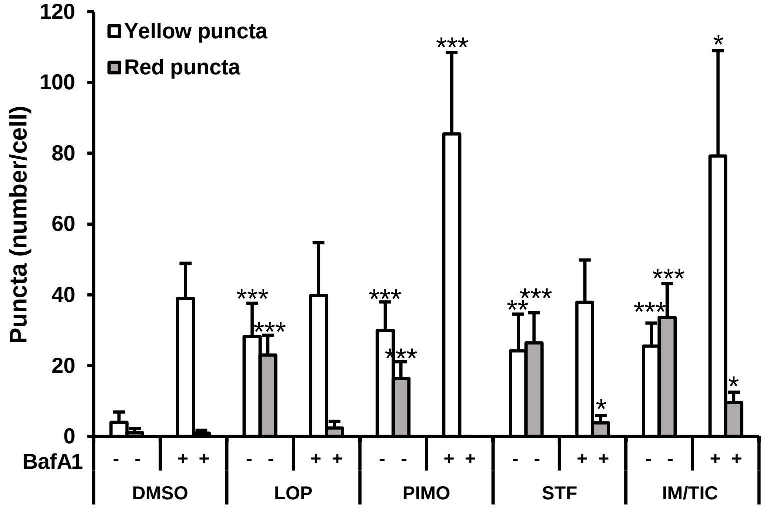

Supplementary Figure Legends

Supplementary Figure S1. ABT-737 in combination with etoposide induces autophagy-independent cell death in MZ-54 cells.

MZ-54 WT, *ATG5* KO and *ATG7* KO cells were treated with indicated concentrations of ABT-737/etoposide for 48 hours. Cell death was assessed by measuring the PI uptake as fraction of total nuclei determined by Hoechst counterstaining using high-content fluorescence microscopy. Mean and SEM of three independent experiments performed in triplicate are shown. ABT = ABT-737, ETO = etoposide.

Supplementary Figure S2. ABT-737 in combination with etoposide induces autophagy-independent cell death of MZ-54 in a time-dependent manner.

MZ-54 cells were treated with the indicated concentrations of ABT-737/etoposide for 24, 48 and 72 hours. Cell death was assessed by measuring the PI uptake as fraction of total nuclei determined by Hoechst counterstaining using high-content fluorescence microscopy. Mean and SEM of three independent experiments performed in triplicate are shown. Significances are calculated *versus* WT cells. * $p<0.05$. ABT = ABT-737, ETO = etoposide.

Supplementary Figure S3: Loperamide, pimozide and STF-62247 induce autophagy-dependent cell death in several GBM cell lines.

LN-229 WT, LN-229 *ATG7* KO, U343 WT and U343 *ATG5* KO cells were treated with the indicated concentrations of loperamide (**A**), pimozide (**C**) and STF-62247 (**E**) for 48 hours (A, C) or 72 hours (E) or with 20 µM loperamide (**B**), 12.5 µM pimozide (**D**) or 30 µM STF-62247 (**F**) for the indicated time points. Cell death was assessed by measuring PI uptake as fraction of total nuclei determined by Hoechst counterstaining using high-content fluorescence microscopy. Data are presented as mean and SEM of three to five

independent experiments performed in triplicate. Significances are calculated against WT cells. * $p<0.05$, ** $p<0.01$, *** $p<0.001$.

Supplementary Figure S4. Rapamycin does not induce cell death in MZ-54 cells.

MZ-54 WT, *ATG5* KO and *ATG7* KO cells were treated with indicated concentrations of rapamycin for 24 hours (**A**), 48 hours (**B**) or 72 hours (**C**). Cell death was assessed by measuring the PI uptake as fraction of total nuclei determined by Hoechst counterstaining using high-content fluorescence microscopy. Data are presented as mean and SEM of three independent experiments performed in triplicate.

Supplementary Figure S5. Effect of Loperamide, pimozide and STF-62247 on ROS formation.

A MZ-54 WT and *ATG7* KO cells were pre-treated with 200 µM trolox, 2.5 mM reduced GSH or 100 µM α-Toc for one hour prior to treatment with 17.5 µM loperamide, 15 µM pimozide, 40 µM STF-62247 or 0.5 µM RSL3 for 48 hours. Cell death was assessed by measuring the PI uptake as fraction of total nuclei determined by Hoechst counterstaining using high-content fluorescence microscopy. Significances of samples treated in the presence of ROS scavengers are calculated against the corresponding samples treated in the absence of ROS scavengers. **B** MZ-54 WT and ATG7 KO cells were treated with 17.5 µM loperamide, 15 µM pimozide or 40 µM STF-62247 for 24 hours, with 0.5 µM RSL3 for two hours (left panel) or with 0.6 µM RSL3 for 6 hours (right panel). ROS production was determined by FACS analysis of the viable cell population using the fluorescent dye CM-H2DCFDA and is shown as fold change compared with control. Data are presented as mean and SEM of three to five independent experiments performed in triplicate. Significances are calculated against untreated samples of the corresponding cell line. * $p<0.05$, ** $p<0.01$, *** $p<0.001$. UT = untreated, LOP = loperamide, PIMO = pimozide, STF = STF-72247.

Supplementary Figure S6: Loperamide, pimozide and STF-62247 induce autophagy of MZ-54 in a time-dependent manner.

MZ-54 cells were treated with 20 µM IM/100 µM TIC, 17.5 µM loperamide, 15 µM pimozide and 40 µM STF-62247 for the indicated time points followed by detection of LC3B and vinculin protein levels by Western blotting with vinculin as loading control. UT = untreated, IM = imipramine hydrochloride, TIC = ticlopidine, LOP = loperamide, PIMO = pimozide, STF = STF-62247.

Supplementary Figure S7: Loperamide, pimozide and STF-62247 induce autophagy in LN-229 and U343 GOS-3 cells.

LN-229 WT and *ATG7* KO cells as well as U343 WT and *ATG5* KO cells were treated with 20 µM loperamide (**A**), 12.5 µM pimozide (**B**) and 30 µM STF-62247 (**C**) for 24 hours followed by detection of ATG7, ATG5, LC3B and vinculin protein levels by Western blotting with vinculin as loading control. UT = untreated, LOP = loperamide, PIMO = pimozide, STF = STF-62247.

Supplementary Figure S8. Loperamide, pimozide and STF-62247 enhance the autophagic flux of MZ-54 cells.

A MZ-54 cells stably expressing mRFP-GFP-LC3B were treated with 20 µM IM/100 µM TIC, 15 µM loperamide, 15 µM pimozide and 40 µM STF-62247 for 30 hours in the absence or presence of 40 nM BafA1 followed by fluorescence microscopy of mRFP-GFP-LC3B and DAPI-stained nuclei. Scale bar = 30 µm. **B** Quantification of yellow and red puncta was performed for three independent experiments with three to five images per sample. Significances are calculated against the corresponding DMSO sample in the presence or absence of BafA1. * $p<0.05$, ** $p<0.01$, *** $p<0.001$. LOP = loperamide, PIMO = pimozide, STF = STF-62247, IM = imipramine hydrochloride, TIC = ticlopidine.
